# Beneficiaries or Charity: The Influence of the Source of Acknowledgments

**DOI:** 10.3389/fpsyg.2021.602410

**Published:** 2021-06-24

**Authors:** Feng Wenting, Xu Yuanping, Wang Tao

**Affiliations:** ^1^Department of Gemstone, Gemmological Institute, China University of Geosciences, Wuhan, China; ^2^Research Center for Psychological and Health Sciences, Marxism School, China University of Geosciences, Wuhan, China; ^3^Department of Marketing, Economics and Management School, Wuhan University, Wuhan, China

**Keywords:** acknowledgment letter, thankers, subsequent donation desire, entitativity, charitable organizations

## Abstract

This study employs entitativity theory to explore how acknowledgment letters from various thankers (e.g., a group of beneficiaries, a charity) influence donors’ subsequent donation desires. This empirical research consists of three experiments. Study 1 reveals that an acknowledgment letter from a group of beneficiaries elicits more favorable subsequent donation desires than an acknowledgment letter from a charity. To shed light on the psychological mechanism underlying this effect, Study 2 shows that a categorical appellation can enhance the effects of an acknowledgment letter sent by a charity. Study 3 reveals that the influence of the thanker is stronger under no external pressure conditions (than under external pressure). The current study offers insightful suggestions for the management and administration of charities.

## Introduction

Charities usually need a long time to recoup the costs of acquiring new donors, and currently, numerous charities face a crisis of donor loyalty (Sargeant and Woodliffe, 2007). Many new donors never give again, as a result of which the funds spent on publicity and recruitment are wasted (Sargeant and Kaehler, 1998). In addition, charities miss the large subsequent fundraising of these lost donors. For example, a 10% decrease in attrition can elicit a 200% improvement in project value (Sargeant, 2001).

Consequently, charities have focused on the retention of donors (Johnson et al., 2014; Merchant et al., 2010). Previous studies have shown that communications and interactions can influence donors’ subsequent donation desires (Gundlach et al., 1995; Mathur, 1996; Bennett, 2009). To strengthen their relationships with donors, non-profits should build strong bonds with them by providing acknowledgments (Merchant et al., 2010), which play an essential role in donors’ future donation desires (Bennett, 2006).

However, in modern life, donors often receive acknowledgment letters from various thankers (a group of beneficiaries or a charity) expressing gratitude for their donations. A group of beneficiaries comprises people who receive help, and a charity comprises people who devote themselves to philanthropy. Do subsequent donation desires of donors vary with the type of thanker acknowledging a donation? Previous studies have mainly focused on the influence of moderating variables, such as occasions (public or private) (Kotler and Lee, 2005), charitable events (Fisher and Ackerman, 1998), content (Newman and Shen, 2012), and individual characteristics (Winterich et al., 2013), on acknowledgment letters. Previous studies are failed to provide a suitable answer to this question, and this gap may exist partially because these studies have not distinguished between thankers (Bennett, 2006; Merchant et al., 2010).

Therefore, the current research explores the influence of thanker type on donors’ subsequent donation desires according to entitativity (the degree to which a social group is perceived as an entity) theory (Campbell, 1958). Study 1 shows that thanker type influences donors’ subsequent donation desires and constructs an integrative mechanism for this effect. Study 2 demonstrates that a categorical appellation can improve the effects of an acknowledgment letter sent by a charity. Study 3 reveals that the influence of thanker type on subsequent donation desires is significant only under no external pressure conditions. Since the information on whether a donation has reached its intended beneficiaries may vary with thanker type, the researcher informed all the donors that the beneficiaries received the donations to exclude this effect. The current research also included several tests to exclude the matching effect between the content of the acknowledgment letter and the thanker (better for a group of beneficiaries or better for a charity).

## Literature Review

### Gratitude

Gratitude is a feeling that occurs in interpersonal exchanges when one person acknowledges receiving a valuable benefit from another. It is typically associated with the perception that one has received a personal benefit that was not intentionally sought, deserved, or earned but rather because of the good intentions of another person (Emmons and McCullough, 2003; Emmons, 2013; Bernabé-Valero et al., 2019a,b). Gratitude is a natural emotional reaction and quite likely a universal tendency to respond positively to another’s benevolence (Emmons and Stern, 2013). Its presence is felt and expressed in various ways by virtually all people of all cultures worldwide (Emmons and Stern, 2013).

Gratitude appears to be one of the most critical components of a good life. The trait of gratitude is correlated with a host of well-being variables (McCullough et al., 2002; Watkins et al., 2003; Krause et al., 2015), and experimental studies have consistently found that gratitude exercises enhance subjective well-being (Emmons and McCullough, 2003; Watkins, 2014; Watkins et al., 2015; Solom et al., 2016). The practice of gratitude can have dramatic and lasting positive effects in a person’s life (Emmons and Stern, 2013). Grateful people are rated by others as more agreeable, helpful, outgoing, optimistic, and less depressed, envious, and neurotic (McCullough et al., 2002). Grateful people tend to experience more frequent positive emotions, such as contentment, happiness, and hope, as well as fewer negative emotions (Watkins et al., 2003; Krause et al., 2015, 2017).

Second, gratitude supports the formation and strengthening of supportive relationships between people (Watkins, 2004; Emmons, 2007; Froh et al., 2011). Gratitude is related to a wide range of adaptive social outcomes, including quality relationships, generosity, compassion, empathy, and learning (McCullough et al., 2002; Wood et al., 2010; Bernabé-Valero et al., 2019a). Gratitude may increase trust in others, aid social integration, and help people contribute to the collective (Deci and Ryan, 2000; Dunn and Schweitzer, 2005). Research indicates that gratitude is strongly related to social functioning because it focuses people on self-improvement and helps them maintain and build strong, supportive social ties (Emmons and McCullough, 2004; Watkins, 2014).

Finally, gratitude is a vital ingredient in prosocial behavior. Gratitude is a moral affect that serves as a moral barometer for beneficiaries by signaling the value of the relationship with the benefactor for the gift bestowed upon them, as a moral reinforcer by increasing the probability that the benefactor will bestow gifts again in the future, and as a moral motive by spurring beneficiaries to respond prosocially toward the benefactor or other people (McCullough et al., 2001). Other studies have also produced convincing evidence in support of the moral motive function of gratitude (Bartlett and DeSteno, 2006; Tsang, 2006, 2007). Grateful people are more helpful, supportive, forgiving, and empathic toward others (McCullough et al., 2001, 2002). Feeling grateful causes people to respond prosocially to benefactors (Bartlett and DeSteno, 2006; Tsang, 2006, 2007) and unrelated others (Bartlett and DeSteno, 2006; Nowak and Roch, 2007).

### Acknowledgment Letters

Non-profit organizations use recognition to encourage prosocial behavior (Kotler and Lee, 2005; Grace and Griffin, 2006) and to cultivate relationships with donors (Merchant et al., 2010), and appreciation can be formal or informal, public (e.g., names listed in newsletters; Kotler and Lee, 2005) or private (appreciation expressed in personal conversations or an acknowledgment letter to a donor; Merrill, 2005). The psychology literature shows that an acknowledgment letter can motivate subsequent donation behavior through the positive emotions that emerge in social interactions (Keltner and Haidt, 1999; McCullough et al., 2001). Gratitude is positive feedback that donors have helped recipients, which should lead donors to feel competent and experience a sense of being capable of achieving a good outcome (Penner et al., 2005). Furthermore, gratitude provides explicit evidence that helpers’ efforts, rather than being devalued or rejected, are important to beneficiaries, encouraging further helping behavior (Grant and Gino, 2010). When a beneficiary expresses gratitude, the benevolence of the benefactor is reinforced, and thus, the benefactor becomes more likely to perform such benevolent behaviors in the future. Conversely, ingratitude leads benefactors to experience anger and resentment and to reduce their willingness to engage in prosocial behavior in the future (McCullough et al., 2001).

However, this positive effect is affected by some moderating variables, such as occasions (public or private) (Kotler and Lee, 2005), charitable events (Fisher and Ackerman, 1998), content (Newman and Shen, 2012), and individual characteristics (Winterich et al., 2013). For example, Newman and Shen (2012) find that small gifts attached to thank-you letters, such as pens, coffee mugs, and handbags, hurt subsequent donations. Kotler and Lee (2005) discover that the external rewards provided by gratitude on public occasions, such as social status and reputations (Belk, 1995), hurt the internal motivation for donation behaviors (Nisbett and Valins, 1972; Lepper, 1981). Private gratitude inspires spiritual satisfaction and positive emotions (McCullough et al., 2001; Colarusso, 2006), which can promote subsequent donation behaviors (Grace and Griffin, 2006). In addition, the characteristics of charitable events are also important factors. Only when donors consider charitable events to be important and meaningful can gratitude promote donation behaviors (Fisher and Ackerman, 1998). Finally, Prince and File (1994) find that some donors do not respond positively after receiving thanks. Gratitude works only for people with low moral identity internalization and high moral identity symbolization (Poole et al., 2011; Winterich et al., 2013).

Previous studies have not explored an important factor: the thanker sending acknowledgment letters. Therefore, the current research analyzes the influence of thanker type on donors’ subsequent donation desires according to entitativity theory.

### Entitativity Theory

Entitativity is the extent to which a social group is perceived to be an entity, not a cluster of loose individuals (Campbell, 1958). Mainstream research argues that there are two key antecedents of entitativity: similarity and dynamics (Wilder and Simon, 1998). Similarity is the degree to which the objects in a group share some property or properties that elicit a categorical thinking style. The group dynamics literature (Cartwright and Zander, 1960) takes a group to be a small collection of interacting individuals with a common object, which leads to a dynamic thinking style. Further, research demonstrates that a categorical group achieves entitativity through the similarity of its members and that a dynamic group obtains entitativity through a common goal and uniform behavior (Wilder and Simon, 1998; Rutchick et al., 2008).

The approach of a group achieving entitativity will influence people’s cognition of the group (Rutchick et al., 2008; Clark and Thiem, 2015). When a group achieves entitativity through a dynamic antecedent and elicits a dynamic thinking style, the cognitive representation of this group is the common goal (Rutchick et al., 2008). For example, a jury, as a task group as defined by Lickel et al. (2000), is a dynamic group that achieves entitativity through the common purposes of the group. Additionally, the cognitive representation of a jury is the purpose of the group, which is the trial. Moreover, dynamic groups that are active and goal-directed elicit perceptions of intentionality (Morris et al., 2001). People perceive the intentionality of a dynamic group as guiding group action to achieve a common goal (Kashima, 2004; Effron and Knowles, 2015). According to Abelson et al. (1998), the activities of a dynamic group trigger inferences that it engages in actions for the sake of a common goal.

If similarity is a criterion for membership, then a categorical group results (Wilder and Simon, 1998). However, a categorical group prompts a categorical thinking style among observers (Rutchick et al., 2008) that is unrelated to the intentionality of the group. Under this condition, people envision not the actual existence of the group but a prototype (Brewer and Harasty, 1996) or a homologous sample (Smith and Medin, 1981). For example, Chinese Americans are a categorical group whose cognitive representation is a virtual individual (a prototype) with the essential traits of this group (American citizens of Chinese heritage).

### The Influences of Thanker Type

However, the characteristic of an actor is a critical factor in people’s attributions about a shared action (Jones and Davis, 1965). A group of beneficiaries is people receiving bits of help that achieve entitativity through a categorical antecedent and elicits a categorical thinking style. The people in this group have common characteristics (receiving help). However, a charity is a group of people devoting themselves to philanthropy that achieves entitativity through a dynamic antecedent and triggers a dynamic thinking style. They aim to achieve the common goal of charity. Therefore, variations in thanker type result in different cognitive representations and attributions.

As a categorical group, a group of beneficiaries prompts a categorical thinking style (Rutchick et al., 2008). The cognitive representation of the group is a prototype (a virtual beneficiary) that encourages people to attribute an interpersonal nature to the action of the group. According to interpersonal interactions, when people benefit from others’ gifts or favors, they acknowledge these efforts (Weiner, 1985). In this condition, donors should assume that the purpose of an acknowledgment letter is to deliver thanks.

In contrast, a charity triggers a dynamic thinking style (Rutchick et al., 2008). The cognitive representation of this group is the common goal (Morris et al., 2001). In this condition, the activities of this group will trigger inferences that it engages in actions for the sake of a common goal (Kashima, 2004). As a result, donors should be prone to infer that acknowledgment letters are issued for other purposes (for example, raising money or advertising for the common goal of philanthropy).

If the antecedents by which a source (beneficiaries, charities) of an acknowledgment letter achieves entitativity vary, the attributions for the acknowledgment letter may vary, which may alter the influence on donors’ subsequent donation desires. People attribute an action endogenously if the purpose appears to be satisfying in itself; this attribution can suggest subjective freedom and lead to positive emotions (Kruglanski, 1975). However, people attribute an action exogenously if it appears to have a further end; this attribution implies compulsion and generates negative emotions (Kruglanski, 1975). Therefore, when donors receive acknowledgment letters from beneficiaries, donors should assume that the purpose of the acknowledgment letters is to deliver thanks. As a result, they should be more likely to view the beneficiaries’ acknowledgment letters endogenously, generating positive emotions and promoting subsequent donation desires. However, when donors receive acknowledgment letters from a charity, donors are prone to assume that acknowledgment letters from charities signal intentionality, such as attracting further donations to achieve the common goal of the organization, and not just to express thanks; this assumption leads to exogenous attributions and negative emotions. Under this condition, an acknowledgment letter cannot convey innate positive emotions of thanks, which will inhibit subsequent donation desires.

H1a: An acknowledgment letter from a group of beneficiaries elicits more favorable subsequent donation desires than an acknowledgment letter from a charity.
H1b: The antecedent of entitativity mediates the relationship between thankers and subsequent donation desires in the mediation analysis.

### The Moderating Role of a Situational Factor

The actor is not the only influence on attribution; the external situation can also affect inferences (Krull, 1993; Gilbert et al., 1998). When an action is under external pressure, people ignore the actor when making an attribution for the action (Kelley, 1973; McClure, 1998). The reason is that when an action is under strong external pressure, people will think that the action occurs because of external stimuli, not the actor, which means that regardless of the traits of the actor, the same action would still occur. Therefore, if the action is under external pressure, then people ignore the actor and attribute the action to the external stimuli (Ross et al., 1977). Thus, they are likely to make an exogenous attribution and negatively evaluate the action (Kruglanski, 1975). However, if an action is not under an external pressure condition, the characteristic of the actor is a critical factor in people’s attribution for the action (Jones and Davis, 1965).

In the current research, the situational factor (no external pressure, external pressure) moderates the relationship between thanker type and subsequent donation desires. Under no external pressure conditions, donors assume that the purpose of the letters from the beneficiaries is to convey appreciation and that the charity issues the letters for other purposes. As a result, more positive emotions and higher subsequent donation desires are generated by acknowledgment letters from the beneficiaries than the letters from the charity. Under external pressure conditions, regardless of whether the acknowledgment letter is issued by the beneficiaries or the charity, it will trigger inferences that the thanker engages in the action for the sake of the external stimuli. As a result, both types of thanker elicit a dynamic thinking style and do not significantly affect subsequent donation desires.

H2: Under no external pressure conditions, an acknowledgment letter from a group of beneficiaries triggers higher subsequent donation desires than an acknowledgment letter from a charity. Under external pressure conditions, subsequent donation desires do not differ regardless of whether the acknowledgment letter is issued by a group of beneficiaries or a charity.

## Materials and Methods

### Study 1

Study 1 tests the basic premise that an acknowledgment letter from a group of beneficiaries elicits more favorable subsequent donation desires than an acknowledgment letter from a charity, which supports H1. This study adopts a between-subjects design (beneficiaries, charity). Since the information on whether a donation has reached its intended beneficiaries may vary with thanker type, the researcher informed all the donors that the beneficiaries had received the donations to exclude this effect.

#### Participants

Based on the calculation method adopted by Cohen (1977) (the effect size *d* = 0.5 and the expected power = 0.80), G^∗^Power 3.1 software was used to determine the sample size (more than 128 people). Therefore, this study recruited 150 donors from a public university in China to complete a series of experiments. The participants were randomly assigned to one of two groups (beneficiaries, charity). The final sample size was 142 (female 47.89%, age from 18 to 38, *M* = 23.53, *SD* = 4.37; *n*_*beneficiary*_ = 70, *n*_*charity*_ = 72).

#### Pretest

To ensure that the charity employed in Study 1 was considered a dynamic group and elicited a dynamic thinking style, an initial group of participants from the internet (*n* = 36, female 55.56%, age from 19 to 41, *M* = 24.64, *SD* = 5.28) was recruited to read the name of the charity (Hope Project). These participants then identified the three most important factors of their assigned group. According to Rutchick et al. (2008), participants should report more verb information than noun information when they consider a charity (Hope Project) to be a dynamic group that employs a dynamic thinking style. The results revealed that the participants reported verb information more frequently than noun information [*M*_*verbal*_ = 2.28, *SD* = 0.57, *M*_*noun*_ = 0.72, *SD* = 0.57, *t*(35) = 8.24, *p* < 0.001, *d* = 2.79], which verified the viability of the charity employed in Study 1.

#### Procedure

In the main experiment, the researchers raised money from each donor (all donations came from donors) at a university for beneficiaries (children living in remote mountainous areas) who had financial problems. The basic information of the recipients appeared on a computer. Then, the experimenter recorded the information of 150 donors, including their name, address, email address, and cell phone number, and randomly assigned them to either the beneficiaries group or the charity group. After 10 days, different acknowledgment letters were sent to the donors based on their group. The participants in the beneficiaries group received acknowledgment letters from the beneficiaries (the children), and the participants in the charity group received acknowledgment letters from the charity (Hope Project). The contents of the letters were identical: “Thank you very much for your help. It is very important and meaningful. Best wishes to you!”

Subsequently, the experimenter sent email invitations to the donors to participate in an online survey. Since the letters from beneficiaries were more likely to indicate that “the donations reached the beneficiaries in need” than the letters from charities, the researcher informed the donors that all the beneficiaries had received the donations, along with a photo of the beneficiaries receiving the donations in the email. Then, the online survey asked whether the donors received a thank-you letter (yes or no) and questioned them about the antecedent of entitativity of the thanker (seven-point scales, 1 = “the group acts as an entity through a common trait.” 7 = “the group acts as an entity through a common goal”; Rutchick et al., 2008). Then, the survey indicated that the charity would hold another donation drive in 10 days and asked whether the participants were willing to donate again using seven-point scales (1 = no desire, 7 = surely donate). The key questions appeared interspersed with irrelevant items about the impression of the beneficiaries, donation reasons, and activity suggestions, among others. Finally, the donors identified the thanker who sent the acknowledgment letter, reported the extent to which the beneficiaries were believed to have received the donations (seven-point scales, 1 = 0%, 7 = 100%), and guessed the purpose of this online survey. To rule out the matching effect between the content of the acknowledgment letter and the thanker, the donors reported the matching degree of the content style and the thanker (seven-point scales, 1 = Very mismatched, 7 = Very matched). For Study 1, an actual charitable activity took place 10 days later, with reminder e-mails sent to each donor 1 day in advance. The experimenter recorded which donors provided second donations and the amount of the donation (US $). After completing all the experiments, the researcher informed the participants of the actual purpose of Study 1 and refunded all donations.

#### Results

Five participants did not respond. Three participants identified the author of the acknowledgment letter incorrectly. No respondents correctly guessed the purpose of the online survey. The beneficiaries group and the charity group did not differ on the extent to which they believed the beneficiaries had received the donations [*M*_*beneficiaries*_ = 5.64, *SD* = 1.04, *M*_*charity*_ = 5.50, *SD* = 0.90; *t*(140) = 0.88, *p* = 0.382, *d* = 0.14]. The two groups also did not appear to significantly differ in the matching degree of the content style and the thanker [*M*_*beneficiaries*_ = 5.03, *SD* = 0.92, *M*_*charity*_ = 5.19, *SD* = 0.87; *t*(140) = 1.11, *p* = 0.269, *d* = 0.18].

Significant differences in the antecedents of entitativity and subsequent donation desires arose between the beneficiary group and the charity group. First, the results revealed that the participants in the beneficiaries group were more likely to elicit a categorical thinking style than those in the charity group [*M*_*beneficiaries*_ = 4.67, *SD* = 0.97, *M*_*charity*_ = 3.22, *SD* = 0.83; *t*(140) = 9.57, *p* < 0.001, *d* = 1.61]. Second, the participants who received the acknowledgment letter from the beneficiaries expressed higher subsequent donation desires than the participants who received the acknowledgment letter from the charity [*M*_*beneficiaries*_ = 5.21, *SD* = 0.96, *M*_*charity*_ = 3.31, *SD* = 0.97; *t*(140) = 11.75, *p* < 0.001, *d* = 1.97], supporting H1a.

A large distinction also occurred between the beneficiaries group and the charity group on subsequent donation behavior (χ = 12.54, *df* = 1, *p* < 0.05), such that the respondents in the beneficiaries group were more likely to donate again (45.71%) than those in the charity group (18.06%). Second, the large difference showed that the donors who received acknowledgment letters from the beneficiaries were more likely to donate more than those who received acknowledgment letters from the charity [*M*_*beneficiaries*_ = 3.66, *SD* = 1.04, *M*_*charity*_ = 2.00, *SD* = 0.58; *t*(43) = 5.41, *p* < 0.001, *d* = 1.97], in support of the predicted main effect in real donations.

##### Mediation analysis

We further tested the mediating role of the antecedent of entitativity in the relationship between thanker type and subsequent donation desires. As predicted, the results of a bootstrapping analysis using PROCESS model 4 (with 5000 bootstrapping resamples; see Hayes, 2013) found a significant indirect effect of thanker type on subsequent donation desires through the antecedent of entitativity (95% confidence interval β = 1.26; *CI* = 0.98–1.56). The results revealed that the effect of thanker type on subsequent donation desires was mediated by the antecedent of entitativity, which supported H1b (see Table 1).

**TABLE 1:** Study 1: mediation results.

Model Path Estimates				

Indirect Effect (with Bootstrap 95% Confidence				
Interval and Standard Errors)				

Effect		LL95% CI	UL95%	CI SE
Thankers—Entitativity	1.45	1.15	1.75	0.15
Entitativity—Donation Desires	0.87	0.76	0.98	0.05
Thankers—Entitativity—Donation Desires	1.26	0.98	1.56	0.15
Thankers—Donation Desires	0.65	0.41	0.89	0.12

*All coefficients are unstandardized, and different thankers were coded as 1 = the beneficiaries group or 0 = the charity group.*

The finding that thanker type affects donors’ subsequent donation desires supports H1. The results also provide strong evidence supporting the mediating function of the antecedent of entitativity. Therefore, charities should offer acknowledgment letters from beneficiaries when possible to maintain relationships with their donors.

### Study 2

Most social groups inherently invoke one type of entitativity, but in principle, people can form categorical or dynamic groups. If people concentrate on the dynamic characteristics of a group, then they emerge with a dynamic thinking style, but if people focus on the similarity of a group, then they employ a categorical thinking style (Rutchick et al., 2008). Diverse entitativity perspectives on a social group thus stem from emphasizing different group factors. Therefore, a charity can shift donors’ attributions for the acknowledgment letters by stressing certain traits. Using a categorical appellation (a group of people with philanthropic ideals) in the acknowledgment letter can evoke a categorical thinking style and improve donors’ subsequent donation desires.

To shed light on the psychological mechanism underlying this effect, Study 2 examines whether a categorical appellation can enhance the effects of an acknowledgment letter sent by a charity. First, this study employs a categorical style to recreate the appellation of the charity in the acknowledgment letter (categorical name, a group of people with philanthropic ideals), following Rutchick et al. (2008). Second, to eliminate the distinction of language formation, this study also employs a dynamic appellation with a similar formation (dynamic name, a group of people aiming at philanthropic ideals) and compares this dynamic appellation to the innate appellation (innate name, Charity Federation). Finally, this study uses cause-related marketing as a background to expand the practical significance and external validity of the main effect. Study 2 adopts a between-subjects design (beneficiaries, categorical name, dynamic name, innate name).

#### Participants

Based on the calculation method adopted by Cohen (1977) (the effect size *f* = 0.25 and the expected power = 0.80), G^∗^Power 3.1 software was used to calculate the planned sample size (more than 179 people). Therefore, Study 2 recruited 210 donors from a public university in China to complete a series of experiments. The participants were randomly assigned to 1 of 4 groups (beneficiaries, categorical name, dynamic name, innate name). The final sample size was 196 (female 43.37%, age from 19 to 34, *M* = 23.34, *SD* = 4.01; *n*_*beneficiaries*_ = 47, *n*_*categorical name*_ = 49, *n*_*dynamic name*_ = 50, *n*_*innate name*_ = 50).

#### Pretest

To determine whether manipulating the appellation of the charity elicited different thinking styles, a pretest required an initial group of participants from the internet (*n* = 96, female 52.08%, age from 17 to 33, *M* = 23.46, *SD* = 4.43, *n*_*categorical name*_ = 31, *n*_*dynamic name*_ = 33, *n*_*innate name*_ = 32) to read the charity name in Study 2, with appellations constructed categorically (a group of people with philanthropic ideals), dynamically (a group of people aiming at philanthropic ideals), or innately (Charity Federation). These participants then identified the three most important factors of their assigned group. The results revealed that the participants in the categorical group reported noun information more frequently than verb information [*M*_*noun*_ = 2.23, *SD* = 0.50, *M*_*verb*_ = 0.77, *SD* = 0.50, *t*(30) = 8.13, *p* < 0.001, *d* = 2.97]. The participants in the dynamic group [*M*_*verb*_ = 2.03, *SD* = 0.77, *M*_*noun*_ = 0.97, *SD* = 0.77, *t*(32) = 3.96, *p* < 0.001, *d* = 1.40] and innate group [*M*_*verb*_ = 2.19, *SD* = 0.54, *M*_*noun*_ = 0.81, *SD* = 0.54, *t*(31) = 7.27, *p* < 0.001, *d* = 2.61] reported more verb information than noun information. Therefore, the appellations used for a charity in an acknowledgment letter affects thinking style.

#### Procedure

Study 2 was conducted under the setting of selling postcards (US $1 per card) at a public university in China. The researcher told the participants that all revenues would be donated to the Charity Federation to help the beneficiaries (children living in the remote mountainous area) (used in Study 1). When people agreed to purchase the card, the experimenter recorded their information and invited them to participate in a subsequent online survey. The participants who agreed were randomly assigned to one of 4 groups: the beneficiaries group (children of the mountains), the categorical group (a group of people with philanthropic ideals), the dynamic group (a group of people aiming at philanthropic ideals), or the innate group (Charity Federation). They received acknowledgment letters with relevant appellations after 10 days.

Subsequently, an email was sent to invite the responders to participate in an online survey. The researcher informed the donors that the beneficiaries had received the donations (the same as Study 1). Then, the online survey asked the donors to confirm whether they received a thank-you letter, report the antecedent of entitativity, and state whether they would donate again to another activity 10 days later. They also identified the thanker and reported the extent to which they believed the beneficiaries had received the donations, the matching degree of the content style and the thanker, and their perceptions of the purpose of the survey. After completing all the experiments, the researcher informed the participants of the actual purpose of Study 2 and refunded all donations.

#### Results

Eight participants did not respond. Six participants failed to identify the author of the acknowledgment letter. No participant guessed the true goal of the online survey. The four groups did not differ in the extent to which they believed the beneficiaries to have received the donations [*F*(3,192) = 0.86, *p* = 0.463, *M*_*beneficiaries*_ = 5.40, *SD* = 1.01, *M*_*categorical name*_ = 5.47, *SD* = 0.79, *M*_*dynamic name*_ = 5.24, *SD* = 0.98; *M*_*innate name*_ = 5.22, *SD* = 0.91, η^2^ = 0.01]. The four groups also did not significantly differ in the perceived matching degree between the thanker and the content style [*F*(3,192) = 0.05, *p* = 0.984, *M*_*beneficiaries*_ = 5.11, *SD* = 1.05, *M*_*categorical name*_ = 5.12, *SD* = 0.95, *M*_*dynamic name*_ = 5.18, *SD* = 1.00; *M*_*innate name*_ = 5.14, *SD* = 0.88, η^2^ < 0.001].

There was a significant difference in the antecedent of entitativity among the four groups [*F*(3,192) = 69.41, *p* < 0.001, η^2^ = 0.52]. The participants in the beneficiaries group were more likely to elicit a categorical thinking style (*M*_*beneficiaries*_ = 5.04, *SD* = 1.04) than those in the innate group [*M*_*innate*_ = 3.04, *SD* = 0.73, *t*(95) = 11.03, *p* < 0.001, *d* = 2.23] and those in the dynamic group [*M*_*dynamic*_ = 3.28, *SD* = 0.81, *t*(95) = 9.34, *p* < 0.001, *d* = 1.89]. The participants in the categorical group (*M*_*categorical*_ = 4.94, *SD* = 0.97) were also more inclined to trigger a categorical thinking style than those in the innate group [*t*(97) = 11.06, *p* < 0.001, *d* = 2.21] and those in the dynamic group [*t*(97) = 9.27, *p* < 0.001, *d* = 1.86]. As expected, no significant distinction appeared between the beneficiaries group and the categorical group [*t*(94) = 0.51, *p* = 0.614, *d* = 0.10]. The data also revealed no obvious difference between the innate and dynamic groups [*t*(98) = 1.56, *p* = 0.122, *d* = 0.31].

A large difference in subsequent donation desires of the four groups emerged [*F*(3,192) = 83.56, *p* < 0.001, η^2^ = 0.57]. The participants in the beneficiaries group reported higher donation desires (*M*_*beneficiaries*_ = 5.60, *SD* = 1.01) than those in the innate group [*M*_*innate*_ = 3.42, *SD* = 0.76; *t*(95) = 12.01, *p* < 0.001, *d* = 2.44] and those in the dynamic group [*M*_*dynamic*_ = 3.64, *SD* = 0.69, *t*(95) = 11.15, *p* < 0.001, *d* = 2.27]. The participants in the categorical group also expressed greater donation desires (*M*_*categorical*_ = 5.37, *SD* = 0.97) than those in the innate group [*t*(97) = 11.12, *p* < 0.001, *d* = 2.24] and those in the dynamic group [*t*(97) = 10.20, *p* < 0.001, *d* = 2.06]. As expected, no significant difference in donation desires appeared between the beneficiaries group and the categorical group [*t*(94) = 1.13, *p* = 0.263, *d* = 0.23]. There was also no obvious difference in donation desires of the innate group and the dynamic group [*t*(98) = 1.51, *p* = 0.133, *d* = 0.30].

This empirical evidence reveals that a categorical appellation can improve the influences of an acknowledgment letter from a charity on subsequent donation desires. A dynamic appellation with a similar formation of that of a categorical appellation generates donation desires equal to those of an innate appellation, which removes the potential effects of language formation. These findings further demonstrate the theoretical logic of the main effect and provide a method that charities can use to increase the influence of their acknowledgment letters.

### Study 3

Study 3 tests whether situational factors moderate the relationship between thankers and subsequent donation desires. This study adopts a 2 (no external pressure, external pressure) × 2 (the beneficiary, the charity) between-subjects design.

#### Participants

Based on the calculation method adopted by Cohen (1977) (the effect size *f* = 0.25 and the expected power = 0.80), G^∗^Power 3.1 software was used to calculate the sample size (more than 179 people). Therefore, Study 3 recruited 210 donors from a public university in China to complete a series of experiments. The participants were randomly assigned to 1 of 4 groups. The final sample size was 193 (female 47.67%, age from 18 to 37, *M* = 23.29, *SD* = 4.14; *n*_*no pressure*,beneficiary_ = 46, *n*_*pressure*,beneficiary_ = 50, *n*_*no pressure*,charity_ = 49, *n*_*pressure*,charity_ = 48).

#### Pretest

To confirm that the charity (Volunteers Association) used in Study 3 was considered a dynamic group and elicited a dynamic thinking style, a pretest required an initial group of participants from the internet (*n* = 33, female 51.52%, age from 18 to 36, *M* = 24.39, *SD* = 5.48) to read the charity name in Study 3. These participants then identified the three most important factors of their assigned group. The results revealed that the participants reported verb information more frequently than noun information [*M*_*verb*_ = 2.21, *SD* = 0.60, *M*_*noun*_ = 0.76, *SD* = 0.61, *t*(32) = 6.96, *p* < 0.001, *d* = 2.46], which verified the viability of the charity employed in Study 3.

#### Procedure

The researcher recruited 210 donors from a university in China who donated money to the children (as in Study 1). Afterward, the researcher recorded the information and invited these donors to participate in a subsequent online survey. The participants were arranged into a 2 (no external pressure, external pressure) × 2 (the beneficiaries, the charity) experimental design. The donors in the external pressure group learned that “Charities had the responsibility to give acknowledgments to people who provided help” (charity condition) or that “Beneficiaries would be asked to send acknowledgment letters to people who provided help” (beneficiaries condition). Under the no external pressure condition, no additional information was available to the two groups. After 10 days, the acknowledgment letters from the relevant thankers (beneficiaries condition: children of the mountains, charity condition: Volunteers Association) followed.

Subsequently, the donors received invitations to participate in an online survey. The researcher informed the donors that all the beneficiaries had received the donations (the same as Study 1). Similar to previous studies, this survey asked whether they received the acknowledgment letter, reported the antecedent of entitativity, and assessed their perceptions of the degree of external pressure experienced by the thanker to send the acknowledgment letter (seven-point scales, 1 = “no external pressure,” 7 = “totally under external pressure”). The donors also indicated the possibility of participating in a subsequent donation activity 10 days later. They were also asked to identify the thanker, report the extent to which they believed the beneficiaries had received the donations, the matching degree of the content style and the thanker, and guess the purpose of the online survey. After completing all the experiments, the researcher informed the participants of the actual purpose of Study 3 and refunded all the donations.

#### Results

A total of 200 participants responded to the online survey; seven of them incorrectly identified the author. No one guessed the purpose of the survey correctly. The participants in the external pressure group reported higher external pressure than those in the no external pressure group [*M*_*pressure*_ = 5.59, *SD* = 0.77, *M*_*no pressure*_ = 2.34, *SD* = 0.75, *t*(191) = 24.08, *p* < 0.05, *d* = 4.28]. The beneficiaries group and the charity group did not differ on the extent to which they believed the beneficiaries to have received the donations [*M*_*beneficiaries*_ = 5.34, *SD* = 0.84; *M*_*charity*_ = 5.44, *SD* = 0.84; *t*(191) = 0.82, *p* = 0.413, *d* = 0.12]. The two groups also did not appear to have a significant difference in the matching degree of the content style and the thanker [*M*_*beneficiaries*_ = 5.31, *SD* = 0.79; *M*_*charity*_ = 5.27, *SD* = 0.85; *t*(191) = 0.38, *p* = 0.706, *d* = 0.05].

ANOVA revealed a significant interaction between thankers and situational factors on the antecedent of entitativity [*F*(1,189) = 49.13, *p* < 0.001, η^2^ = 0.13]. Specifically, under the no external pressure condition, the participants who received letters from the beneficiaries were more likely to elicit a categorical thinking style than those who received letters from the charity [*M*_*beneficiaries*_ = 4.85, *SD* = 0.84, *M*_*charity*_ = 3.33, *SD* = 0.80; *t*(93) = 9.02, *p* < 0.001, *d* = 1.85]. However, under the external pressure condition, no obvious difference was found between the beneficiaries group and the charity group [*M*_*beneficiary*_ = 3.46, *SD* = 0.65, *M*_*charity*_ = 3.38, *SD* = 0.70; *t*(96) = 0.62, *p* = 0.534, *d* = 0.12].

The results revealed a predicted interactive effect of thanker type and situational factors on subsequent donation desires [*F*(1,189) = 21.93, *p* < 0.001, η^2^ = 0.08]. Specifically, the donors in the beneficiaries group were more willing to provide future help than those in the charity group under the no external pressure condition [*M*_*beneficiaries*_ = 5.15, *SD* = 0.97, *M*_*charity*_ = 3.88, *SD* = 0.81; *t*(93) = 7.00, *p* < 0.001, *d* = 1.42]. However, no significant difference was found between the charity group and the beneficiaries group [*M*_*charity*_ = 3.86, *SD* = 0.90, *M*_*beneficiary*_ = 3.80, *SD* = 0.90; *t*(96) = 0.38, *p* = 0.708, *d* = 0.07] under the external pressure condition.

Moderated mediation analysis: We further tested the moderating role of situational factors in the relationship between thanker type and subsequent donation desires. The data were submitted to a moderated mediation analysis (using the macro PROCESS, model 8, with 5000 bootstrapping resamples; see Hayes, 2013). The results revealed that the moderating effect of situational factors was significant (95% confidence interval β = 1.32; *CI* = 0.93–1.74). Specifically, the results verified a significant interactive effect of thanker type and situational factors on the antecedent of entitativity (95% confidence interval β = 1.44; *CI* = 1.01 to 1.86). The influence of the antecedent of entitativity on subsequent donation desires was also significant (95% confidence interval β = 0.92; *CI* = 0.81–1.03). These findings indicated that the mediation effect of the antecedent of entitativity was moderated by situational factors (see Table 2).

**TABLE 2:** Study 3: moderating role of a situational factor.

	Model 1		Model 2	
		
	Entitativity (mediator)		Donation desires	
			(with an added mediator)	
		
Independent variables	Coefficient	T	Coefficient	T
Different Thankers	0.09	0.56	–0.01	–0.09
Situational Factor	–0.05	–0.32	0.13	1.13
Thankers*Situational Factor	1.44	6.65***	–0.12	–0.64
Entitativity	–	–	0.92	16.70***

All coefficients are unstandardized; different thankers were coded as 1 = the beneficiaries group or 0 = the charity group, and the situational factor was coded as 1 = the free condition or 0 = the external pressure condition. The indirect effect of the interaction through entitativity was significant for donation desires (β = 1.32; CI [0.9257, 1.7357]). *p < 0.05, **p<0.01, ***p<0.001.

Situational factors moderated the relationship between thanker type and subsequent donation desires, confirming H2. Specifically, under the no external pressure condition, an acknowledgment letter from the beneficiaries triggered a more categorical thinking style and higher subsequent donation desires than an acknowledgment letter from the charity. Under the external pressure condition, no differences appeared between the two groups. Study 3 shows that charities must avoid any hint of external pressure in their acknowledgment letters.

## General Discussion

### Conclusion

The current research explored the influence of thanker type on donors’ subsequent donation desires. Study 1 found that thanker type affects donors’ subsequent donation desires. An acknowledgment letter from a group of beneficiaries elicits more favorable subsequent donation desires than an acknowledgment letter from a charity. Study 2 revealed that using a categorical appellation can improve the effects of an acknowledgment letter from a charity. Study 3 tested the moderating role of situational factors. The results indicate that the positive effect of letters from beneficiaries is significant only under no external pressure conditions.

### Research Contributions

First, the current research has furthered the study of gratitude and verifies the role of gratitude as a moral emotion in promoting subsequent prosocial behavior (McCullough et al., 2001). Previous studies have found that gratitude serves as a moral reinforcer by increasing the subsequent prosocial behavior (Krause et al., 2015). The current research testifies to the influence of the source of acknowledgment letters on the moral reinforcer effect, which expands the research on gratitude.

These initial explorations of the influence of thanker type on subsequent donation desires produce an integrative model of the related mental mechanism and a new theoretical perspective. Previous studies have not distinguished between various thanker types. For example, many studies in this field have shown that the existence of personal relations between individuals can improve relationships and commitment (Sargeant and Woodliffe, 2007; Sargeant, 2014) and elevate trust and affiliation (Bennett and Barkensjo, 2005; Stebbins and Hartman, 2013). Some studies indicate that a single identified person can elicit more effective responses than a group of people, promoting sympathy and donation intentions (Slovic, 2007; Small et al., 2007; Dickert et al., 2011). The current research employed entitativity theory to explain why an acknowledgment letter from a charity differs from a letter from a group of beneficiaries. By testing subsequent donation desires as the dependent variable, this research explored the influence of thanker type and identified an integrative psychological mechanism.

Second, this research shows that the positive effects of expressing gratitude are moderated by thanker type, which potentially enriches the existing relevant literature. Previous studies have found that the positive effects of gratitude are affected by some moderating variables, such as occasions (Kotler and Lee, 2005), contents (Newman and Shen, 2012), and individual characteristics (Winterich et al., 2013). However, if charities want to sustain donor loyalty, the source of the thanks is also crucially important. When the thanker is a dynamic group, such as a charity, donors have lower subsequent donation desires. Furthermore, this research clarifies some boundary conditions by analyzing the effects of a situational factor, finding that the situational factor (no external pressure versus external pressure) verifies the relationship between thanker type and subsequent donation desires.

Third, from a theoretical perspective, an emerging body of research suggests a downstream influence of entitativity on individual behavior (Clark and Thiem, 2015; Effron and Knowles, 2015; Vangrieken et al., 2016). By demonstrating that how a group achieves entitativity (categorical, dynamic) affects subsequent donation desires, the current study reveals the distinction between an acknowledgment letter from a group of beneficiaries and an acknowledgment letter from a charity organization and presents a novel measure for charities to increase subsequent donation desires (employing a categorical appellation). This research also expands the literature on entitativity theory by specifying the behavioral consequences and analyzing the psychological process within a philanthropic context.

### Future Research

Although this study considered how groups achieve entitativity and the influences on subsequent donation desires, it did not analyze the function of individual traits (Louie and Obermiller, 2000; Baek and Reid, 2013). Previous studies suggest that individual traits can affect prosocial performance, so additional research should explore the influence of such traits on boundary conditions. Beneficiaries’ characteristics may also serve as moderators. If beneficiaries show obvious traits that suggest intentionality, will donors still attribute their acknowledgment letters exogenously? Further studies should seek to delineate the influence of individual beneficiary traits on donors’ subsequent donation desires. In addition, one limitation of this research is the possible selection bias: Because this study focused on subsequent donation desires, it analyzed only those participants who initially provided help. However, the results cannot reflect the intentions of those people who initially did not help. Future research can take a further step to explore the reactions of initially non-helping participants to thankers of different types.

## Data Availability Statement

The original contributions presented in the study are included in the article/Supplementary Material, further inquiries can be directed to the corresponding author/s.

## Ethics Statement

The studies involving human participants were reviewed and approved by Center for Mental Health and Science of China University of Geosciences. The patients/participants provided their written informed consent to participate in this study.

## Author Contributions

FW was contributed to responsible for organizing literature, proposing research questions, and analyzing hypothesis logic. XY was contributed to modify the manuscript according to the reviews’ comments. WT was contributed to responsible for collecting and analyzing experimental data. All the authors contributed to the article and approved the submitted version.

## Conflict of Interest

The authors declare that the research was conducted in the absence of any commercial or financial relationships that could be construed as a potential conflict of interest.
